# ‘It’s clever, but is it Art?’

**DOI:** 10.1177/1757913920922274

**Published:** 2020-09-15

**Authors:** Heather Yoeli, Sarah McLusky

**Affiliations:** Durham University, Institute for Medical Humanities, Caedmon Building, Leazes Road, Durham, DH1 1SZ, UK; Durham University, Durham, UK

*In this article, Yoeli and McLusky speak to the fact that while there is no solid definition or explanation for the arts, when in the context of an Arts in Health initiative, it is important to know how the arts are distinguished from arts psychotherapies and the allied health professions.*

We have learned to whittle the Eden Tree to the shape of a surplice-peg,

We have learned to bottle our parents twain in the yolk of an addled egg,

We know that the tail must wag the dog, for the horse is drawn by the cart;

But the Devil whoops, as he whooped of old: ‘It’s clever, but is it Art?’^1^

The Devil is right to ask. In *The Conundrum of the Workshops*,^1^ which both marvels at and worries about the extent of human progress, Kipling reflects the concern prevalent within his era that scientific advancement might one day render obsolete the human affinity for the arts. In evaluating the present-day arts in health (AiH) movement, this worry remains valid.

The question *but is it art?* is vital to the AiH movement because the arts (i.e. music, the visual arts, drama, literature, dance, multimedia, and the diverse and varied emerging new art forms) are what render AiH distinct from the arts psychotherapies (art therapy, psychodrama, music therapy, etc.) and allied health professions (physiotherapy, occupational therapy, nursing, etc.), and in their distinctiveness, uniquely beneficial to health. If a so-called AiH initiative fails to incorporate an element which can truly be considered art, it will fail to deliver those unique health benefits that the arts provide. In recent years, an increasing scarcity of funding opportunities and the continued demand for evidence-based practice has led the AiH movement to work in increasingly close collaboration and partnership with medical and rehabilitative services.^2^ This professionalisation and medicalisation of AiH practice, is inadvertently threatening the extent to which AiH should be considered art.

What *is* art, then? When posed within a worldview which propounds the philosophical virtue of *art for art’s sake*^3^ or of intrinsic value,^4^ this question has little meaning: the arts just *are*, and thereby require no definition, explanation or justification for their existence or utility.^5^ When framed within a neoliberal worldview, in which AiH initiatives are required to provide clinical evidence of their effectiveness to ensure their financial viability, this question nevertheless requires an answer.^6^ Public health policy tends to describe the arts as a means of generating *cultural capital*,^7^ a concept defined as the use of non-economic strategies to promote social mobility and to combat inequality.^8^

The AiH movement embodies the concept of *art for art’s sake* through the ways in which it distinguishes itself from the arts psychotherapies and the allied health professions. Both the arts psychotherapies and allied health professions are essentially task-orientated in their aim to treat symptoms, improve wellbeing, promote coping and produce behaviour change. AiH, however, is fully process-driven, aiming simply to generate a genuine and meaningful artistic experience through painting, singing, ballet or the many other art forms through which AiH operates.

**Figure:**
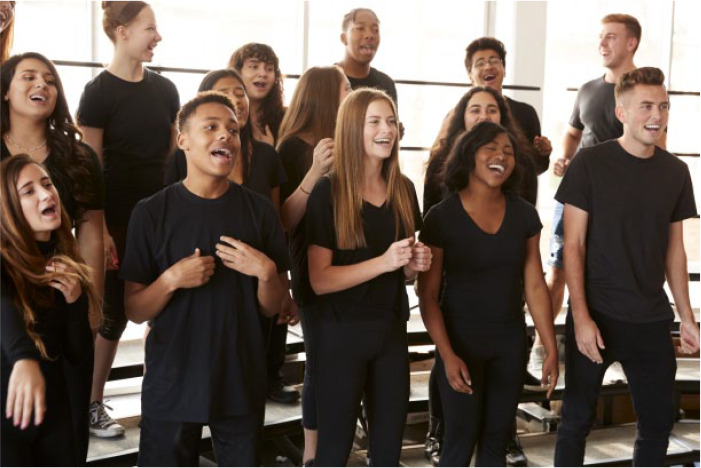


As AiH becomes increasingly drawn into professional healthcare provision, there has been an increasing move to appropriate specific artistic techniques as therapeutic manoeuvres. Some of the vocal exercises used by singers have been employed to teach mindfulness for people experiencing anxiety,^9^ for example, and some ballet movements have been used to promote posture and balance in stroke survivors, often to significant clinical effect. Whenever artistic techniques are removed from their context and stripped of their creative potential in this way, they may lose their meaningful *art for art’s sake* element of creative self-expression – and thereby may no longer be art. AiH most readily preserves its artistic quality when delivered by a dedicated and practicing artist who is able to facilitate its activities from a creative rather than a therapeutic perspective.

The concept of the AiH movement as a means to combat inequality predates the concept of *cultural capital* and is indeed integral to the history and heritage of the AiH movement itself.^10^ Until approximately the turn of the millennium, AiH initiatives were grounded primarily in community-owned, socially engaged and often anti-establishment artistic practices aiming to improve the wellbeing of communities through their advocacy for social and economic inclusion and justice.^11^ To some extent, this AiH activism continues within artistic subcultures such as Forum Theatre,^12^ and within mental health settings.^13^ However, the growing alliance between AiH and mainstream health services has generally encouraged AiH initiatives to advance less subversive and more apolitical views. Whenever art is stripped of the creative freedom needed to contribute to *cultural capital*, it risks no longer being art.

Nevertheless, the presence and role of the practicing artist facilitating AiH activities within healthcare provision remain inherently radical. Healthcare professionals are expected to relate to their patients in highly regulated and tightly boundaried ways. Artistic practitioners, by contrast, are in this regard unconstrained; they are by definition *Outsiders* to mainstream health provision and thereby possess the radical freedom to relate to participants in a creative, equalising and often subversive manner.^14^ Artistic practitioners facilitating AiH activities can radically dignify and empower their participants by elevating each to the status of artistic co-producer, a status far removed from the benevolent, yet distancing, professional gaze.^15^ In so doing, artistic practitioners facilitating AiH activities can enable their participants to create and to co-create their own authentic art. This art, having originated through the participants’ unique and radical relationship and collaboration with the AiH practitioner-artist, carries an inherent social and political message. Through this message, this art thereby makes a valuable contribution to *social capital*.

The role of the facilitator – the practitioner who delivers the AiH interventions – thereby emerges as central to the question facing the contemporary AiH movement. As AiH becomes increasingly integrated within medical or rehabilitative services, this article argues that AiH initiatives will only fulfil their therapeutic potential when facilitated by practicing artists, distinct in their role from healthcare professionals. As AiH practitioners become increasingly aligned with the medicalised thinking of their professional colleagues, they must nevertheless remain true to their heritage as agents of radical empowerment and social change, and as advocates of the inherent value of the artistic process.
